# Supplementary material for: Chromothripsis during telomere crisis is independent of NHEJ, and consistent with a replicative origin
Source: Genome Res. 2019 May;29(5):737–49. doi: 10.1101/gr.240705.118 (PMC6499312; doi:10.1101/gr.240705.118)
Supplement: Supplemental Material [file supp_gr.240705.118_Supplemental_file_1.zip › contigs/annotated_contigs/DB51/contig.2.DB51_length_561_mean_cov_3.69875222816.docx]

**DB51_length_561_mean_cov_3.69875222816**

```
TGAAACCTGCTAGAGGATATTTGCCCAAAACAAGGTAGCTAACGACACTCCATTAAAGACAATGAGCATGAACTGAGACAACTTTTCAG
 >chr6:14407722-14407968 + E=2e-133
GAAAAAAGAACTAATTAAACATTAAACTCTAGTTCCATTATTTAGAATTCTTTTCTGGACATTTTAAATTCAACATGAACATTTACAAT

GATACAATCAGATCGACTCTCCTTTCAGCTAGATATTAAATTATACTTAATGATAACCAAGATT|AGAA|TATTGGAAAGGCCATAGTG
                                                                 >chr6:14965190-1496550
CCGTCATGTGGATCTTTGTAAGGGTTAATGGCTGCTTCTTTGAATCTCAGTGTAGCAGCTGCAGATGACATCAGATTTTCTGCCACATT
9 + E=2e-180
GGCTTAAATCAACACAATGAATCCATCAAGGGGAAGGCCAAGGCACCATTTGGGGCTTACAAGGAAGGACTGGCCAGAGGTGTTCAAAC

AAATGAGATTTCCCCTGCTGTATGAGGACATCTAAAATGTGACCATTTTAGGCATTTTCATAAAACAGTTAACTGCCTTTCTCGCTGAG

AAAAAGACACACAGGACAAACATCAAGAG
```
